# Comparison of absorbed dose extrapolation methods for mouse-to-human translation of radiolabelled macromolecules

**DOI:** 10.1186/s13550-022-00893-z

**Published:** 2022-04-11

**Authors:** Francesco Cicone, David Viertl, Thibaut Denoël, Michael G. Stabin, John O. Prior, Silvano Gnesin

**Affiliations:** 1 Department of Experimental and Clinical Medicine, and Neuroscience Research Centre, PET/MR Unit, “Magna Graecia” University of Catanzaro, Catanzaro, Italy; 2 Nuclear Medicine Unit, University Hospital “Mater Domini”, Catanzaro, Italy; 3 University of Lausanne, Lausanne, Switzerland; 4 Department of Nuclear Medicine and Molecular Imaging, Lausanne University Hospital, Rue du Bugnon 46, 1011 Lausanne, Switzerland; 5 NV5/Dade Moeller, Richland, WA USA; 6 Institute of Radiation Physics, Lausanne University Hospital, Lausanne, Switzerland

**Keywords:** Small animal dosimetry, Allometric equations, Scaling factor, Radiopharmaceuticals, Dose extrapolations, Preclinical models, OLINDA/EXM® 2.0, Biodistribution, Mass scaling, Metabolic time scaling

## Abstract

**Background:**

Extrapolation of human absorbed doses (ADs) from biodistribution experiments on laboratory animals is used to predict the efficacy and toxicity profiles of new radiopharmaceuticals. Comparative studies between available animal-to-human dosimetry extrapolation methods are missing. We compared five computational methods for mice-to-human AD extrapolations, using two different radiopharmaceuticals, namely [^111^In]CHX-DTPA-scFv78-Fc and [^68^Ga]NODAGA-RGDyK. Human organ-specific time-integrated activity coefficients (TIACs) were derived from biodistribution studies previously conducted in our centre. The five computational methods adopted are based on simple direct application of mice TIACs to human organs (M1), relative mass scaling (M2), metabolic time scaling (M3), combined mass and time scaling (M4), and organ-specific allometric scaling (M5), respectively. For [^68^Ga]NODAGA-RGDyK, these methods for mice-to-human extrapolations were tested against the ADs obtained on patients, previously published by our group. Lastly, an average [^68^Ga]NODAGA-RGDyK-specific allometric parameter α_new_ was calculated from the organ-specific biological half-lives in mouse and humans and retrospectively applied to M3 and M4 to assess differences in human AD predictions with the *α* = 0.25 recommended by previous studies.

**Results:**

For both radiopharmaceuticals, the five extrapolation methods showed significantly different AD results (*p* < 0.0001). In general, organ ADs obtained with M3 were higher than those obtained with the other methods. For [^68^Ga]NODAGA-RGDyK, no significant differences were found between ADs calculated with M3 and those obtained directly on human subjects (H) (*p* = 0.99; average M3/H AD ratio = 1.03). All other methods for dose extrapolations resulted in ADs significantly different from those calculated directly on humans (all* p* ≤ 0.0001). Organ-specific allometric parameters calculated using combined experimental [^68^Ga]NODAGA-RGDyK mice and human biodistribution data varied significantly. ADs calculated with M3 and M4 after the application of *α*_new_ = 0.17 were significantly different from those obtained by the application of *α* = 0.25 (both *p* < 0.001).

**Conclusions:**

Available methods for mouse-to-human dosimetry extrapolations provided significantly different results in two different experimental models. For [^68^Ga]NODAGA-RGDyK, the best approximation of human dosimetry was shown by M3, applying a metabolic scaling to the mouse organ TIACs. The accuracy of more refined extrapolation algorithms adopting model-specific metabolic scaling parameters should be further investigated.

**Supplementary Information:**

The online version contains supplementary material available at 10.1186/s13550-022-00893-z.

## Introduction

Radiopharmaceutical development requires experimental testing on laboratory animals, usually small mammals like mice or rats. The biodistribution of a given radiopharmaceutical is most often assessed by killing and dissecting groups of animals at multiple time points after tracer administration. The interest in generating dosimetry data from biodistribution experiments is growing, due to more demanding legal requirements for human translation of new radiopharmaceuticals [1]. Moreover, the assessment of the absorbed dose (AD) to animal organs takes advantage of the recent development of flexible, digital animal phantoms that have been implemented in commercial dosimetry software [2–6].

There are several biological and technical variables that should be considered in animal-to-human translation of biodistribution data and dosimetry. From a biological standpoint, the differences in radiopharmaceutical biodistribution between animals and humans depend on both the radiopharmaceutical under investigation and the animal model used for the experiments. The design phase of a new radiopharmaceutical must ensure the reliability and the translational value of the animal model used for preclinical experiments [7]. Biological variables that may significantly affect radiopharmaceutical biodistribution in animal models include animal stress, pharmacological interference of anaesthetics on hormone secretion and organ function, different cellular expression of targeted molecules, and others [8–10]. When small animals are used for preclinical experiments, the most evident technical difference is represented by the manipulation of dissected organs in the classical biodistribution experiments as opposed to the sequential radioactivity counting in the living body of human subjects, typically obtained from gamma emission quantitative imaging and used to derive organ-specific time-integrated activity coefficients (TIACs) [11–14].

Furthermore, computational aspects are particularly relevant in translational research and were object of extensive previous studies in the pharmaceutical field [15]. Organ sizes and metabolic rates of physiological functions are profoundly different between laboratory animals and humans. These differences are accounted for by allometric equations, usually considering the variable of interest as a dependent function of the body mass [16, 17]. In the radiopharmaceutical field, allometric equations have been applied to animal-to-human AD extrapolations. Two are the most popular methods for extrapolating human dosimetry from small animal data. The simplest method applies the organ TIAC obtained from radiopharmaceutical biodistribution in laboratory animals unmodified to human computational phantoms. In the second method, the source organ TIACs are rescaled by taking into account the relative contribution of single organ masses to the total body weight in both species. More refined methods have been described introducing additional parameters to correct for the different metabolic rates within species, with or without mass scaling [18–25]. However, there is a lack of studies comparing the results of the different equations that were developed for animal-to-human AD extrapolations. The aim of the present study was twofold. Firstly, we compared five reference computational methods for AD extrapolations from mice to humans, using two different radiopharmaceuticals, namely [^68^Ga]NODAGA-RGDyK and [^111^In]CHX-DTPA-scFv78-Fc, that were previously characterized in vitro and in vivo in our centre [24, 25]. In the second instance, for one of these radiopharmaceuticals, i.e. the [^68^Ga]NODAGA-RGDyK, AD extrapolations were compared with ADs obtained in human subjects as part of a former clinical study published by our group [26], and organ-specific allometric scaling factors were retrospectively calculated and discussed.

## Methods

### Radiopharmaceuticals

[^68^Ga]NODAGA-RGDyK is a positron-emitting peptidic radiopharmaceutical targeting the α_v_β_3_ integrin, a specific marker of angiogenesis. Radiolabelling and preclinical evaluation of [^68^Ga]NODAGA-RGDyK were described in [25]. Clinical studies have regarded patients with atherosclerotic plaques and various tumour types including head and neck, oesophageal and brain cancers [26–29]. [^111^In]CHX-DTPA-scFv78-Fc is a gamma-emitting radiopharmaceutical based on a fully human antibody single-chain variable fragment (scFv), cross-reactive with mouse endosialin/tumour endothelial marker 1 (TEM-1), fused to an immunoglobulin crystallizable fragment (Fc). Endosialin/TEM-1 is a marker of tumour-associated stromal fibroblasts and pericytes [30]. Radiolabelling and full preclinical characterization of [^111^In]CHX-DTPA-scFv78-Fc were reported in [24]. This radiopharmaceutical has not yet been translated into clinic.

### Biodistribution experiments and generation of mouse time-integrated activity coefficients

Biodistribution of [^68^Ga]NODAGA-RGDyK was obtained in female, outbred Hsd ICR (CD-1®) mice, killed 10, 30, 60 and 90 min after intravenous radiopharmaceutical injection (3–4 mice per group) [25]. Biodistribution of [^111^In]CHX-DTPA-scFv78-Fc was assessed in female common gamma KO Balb/c mice, grafted with the human TEM-1-positive Ewing’s sarcoma RD-ES. Mice were killed 4, 24, 48 and 96 h after intravenous radiopharmaceutical injection (3–5 mice per group) [24]. For both experiments, organs were harvested and weighed, and radioactivity was counted in a calibrated gamma-counter (Wallac Wizard, PerkinElmer). The list of source organs was reported for each biodistribution experiment separately [24, 25].

At each time point, the activity in each source organ of each single animal was measured and normalized by the total injected activity to obtain the normalized source organ activity (nA). The actual measured data were not corrected for physical decay. For each source organ at each time point, an averaged nA value across all the animals used for the experiment was obtained. For [^111^In]CHX-DTPA-scFv78-Fc, most organs showed effective radiopharmaceutical washout over time. In these organs, a mono-exponential fit extended to infinite beyond the last measured data point was used to derive TIACs by analytical time integration of source organ average normalized time–activity curves (nTACs). In contrast, for organs whose radiopharmaceutical uptake remained constant or increased over the observation period (i.e. spleen, stomach, uterus and ovaries), TIACs were obtained by trapezoidal integration using MATLAB software (Release 2017a, The MathWorks, Inc., Natick, Massachusetts, USA), and a mono-exponential analytical integration to infinite was calculated after the last measure, assuming the radioisotope physical decay.

In case of [^68^Ga]NODAGA-RGDyK, owing to the short half-life of ^68^Ga, all organ TIACs could be obtained by fitting nTACs with a monoexponential function extended to infinite beyond the last data point. Further details on the methodology and theoretical assumptions leading to the generation of TIACs for specific organs are provided elsewhere [24, 25]. As described in [24], to correct for the tumour antigenic sink in tumour-bearing mice injected with [^111^In]CHX-DTPA-scFv78-Fc, the RD-ES tumour TIAC was redistributed into mouse source organ TIACs proportionally to their contribution to the whole-body TIAC (corrected mouse TIACs, TIACs_m,correct_, which, on average, were 16% higher than TIACs obtained without tumour TIAC redistribution [24]).

### Human dose extrapolations from mouse data

Mouse TIACs (TIAC_m_) for [^68^Ga]NODAGA-RGDyK and [^111^In]CHX-DTPA-scFv78-Fc, as well as mouse organ masses m(organ)_m_ and mouse whole-body masses WB_m_, were obtained from Table 1 of [25] and from [24], respectively. In particular, for mouse-to-human AD extrapolations of [^111^In]CHX-DTPA-scFv78-Fc, the TIACs_m,correct_ reported in Supplementary Table 2 of [24] were used. The following five methods were used to obtain human organ TIAC (TIAC_h_) from TIAC_m_, in units of MBq.h/MBq, for both radiopharmaceuticals [18–20, 23]:

**Table 1:** Extrapolated human dosimetry for [^111^In]CHX-DTPA-scFv78-Fc

[^111^In]CHX-DTPA-scFv78-Fc	Average human AD extrapolations (mGy/MBq) (GA Subject)				
Target organ	M1	M2*	M3	M4	M5
Adrenals	4.34E−01	3.15E−01	5.12E−01	3.57E−01	2.07E−01
Brain	1.73E−02	2.43E−02	2.88E−02	4.17E−02	5.91E−02
Breasts	5.01E−02	4.09E−02	6.15E−02	6.73E−02	6.77E−02
Oesophagus	1.56E−01	1.10E−01	1.74E−01	1.45E−01	1.14E−01
Eyes	1.75E−02	2.44E−02	2.90E−02	4.19E−02	5.92E−02
Gallbladder Wall	3.76E−01	2.20E−01	4.09E−01	2.43E−01	2.19E−01
Left colon^†^	2.78E−01	1.07E−01	4.06E−01	1.55E−01	1.01E−01
Small Intestine^†^	5.68E−01	1.55E−01	6.58E−01	1.91E−01	9.98E−02
Stomach Wall^†^	2.38E−01	1.30E−01	2.63E−01	1.56E−01	1.12E−01
Right colon^†^	3.06E−01	1.13E−01	4.54E−01	1.53E−01	1.20E−01
Rectum^†^	2.29E−01	6.61E−02	3.60E−01	1.35E−01	8.60E−02
Heart wall^†^	2.31E−01	1.28E−01	2.80E−01	2.65E−01	1.15E−01
Kidneys^†^	5.77E−01	2.98E−01	9.63E−01	4.58E−01	2.23E−01
Liver^†^	9.23E−01	5.70E−01	8.87E−01	5.68E−01	5.02E−01
Lungs^†^	1.33E−01	1.29E−01	1.78E−01	1.79E−01	1.07E−01
Ovaries^†^	7.31E−01	1.42E−01	7.66E−01	7.14E−01	9.82E−02
Pancreas^†^	3.43E−01	1.82E−01	3.78E−01	2.07E−01	1.57E−01
Prostate	6.29E−02	4.37E−02	9.16E−02	6.30E−02	7.74E−02
Salivary glands	2.15E−02	2.87E−02	3.37E−02	4.77E−02	6.66E−02
Red Marrow^†^	7.28E−02	6.73E−02	1.36E−01	1.23E−01	7.89E−02
Osteogenic cells	8.60E−02	7.58E−02	1.36E−01	1.29E−01	1.20E−01
Spleen^†^	7.14E−01	8.76E−01	7.59E−01	8.84E−01	9.71E−02
Testes	1.88E−02	2.56E−02	2.90E−02	3.82E−02	5.72E−02
Thymus	8.46E−02	6.78E−02	1.05E−01	1.12E−01	8.81E−02
Thyroid	3.88E−02	4.26E−02	5.49E−02	6.58E−02	7.21E−02
Urinary bladder wall	7.62E−02	4.20E−02	1.02E−01	8.27E−02	8.07E−02
Uterus^†^	9.07E−01	1.65E−01	9.53E−01	8.62E−01	9.60E−02
Total body	7.99E−02	5.82E−02	1.01E−01	8.39E−02	8.30E−02
Effective dose (mSv/MBq)	1.96E−01	1.16E−01	2.41E−01	1.64E−01	1.05E−01

Mouse-to-human dose extrapolations were based on time-integrated activity coefficients (TIACs) obtained from biodistribution data on mice, reported in [24]. Human TIACs were calculated for male and female subjects with five different computational methods and used as input in OLINDA/EXM v.2.1 to derive gender average (GA) organ absorbed doses (ADs) and effective doses (ED) according to the ICRP-103 [32]

*Extrapolations according to M2 were already reported in Table 2 of [24]

^†^Indicates source organs

Method 1 (M1): direct mice-to-human extrapolation:

1 $${\text{TIAC}}_{h,M1} \left( {{\text{organ}}} \right) = {\text{TIAC}}_{m} \left( {{\text{organ}}} \right)$$

Method 2 (M2): Extrapolation of TIAC_h_ from mice by the application of a relative mass scaling factor [18]:

2 $${\text{TIAC}}_{h,M2} \left( {{\text{organ}}} \right) = {\text{TIAC}}_{m} \left( {{\text{organ}}} \right) \times \left( {\frac{{m\left( {{\text{organ}}} \right)_{h} /{\text{WB}}_{h} }}{{m\left( {{\text{organ}}} \right)_{m} /{\text{WB}}_{m} }}} \right)$$

where *m*(organ)_h_ are the organ masses and WB_h_ are the whole-body masses considered for each human model (male and female) according to ICRP-89 (WB_h,Male_ = 73 kg; WB_h,Female_ = 60 kg) [31].

Method 3 (M3): Extrapolation of TIAC_h_ by the application of a scaling factor to the biologic component of the murine nTACs; indeed:

3a $$\tau_{b,h} = k_{b} \times \tau_{b,m}$$

where *τ*_b,h_ and *τ*_b,m_ are the organ-specific biologic half-life in human and mice, respectively, and *k*_*b*_ the specie-specific biologic scaling factor expressed by the equation:

3b $$k_{b} = \left( {\frac{{{\text{WB}}_{h} }}{{{\text{WB}}_{m} }}} \right)^{\alpha }$$

where α = 1/4 = 0.25, which assumes that the metabolism of injected drugs or radiopharmaceuticals scales, on average, as the heart and respiratory cycles across species [16]. The analytic formalism of this approach was reported by Sparks and Aydogan [19]. For source organ nTACs fitted with mono-exponential functions, the formalism reduces to the equation:

3c $${\text{TIAC}}_{h,M3} \left( {{\text{organ}}} \right) = \frac{{C_{{m,{\text{organ}}}} \left( {t = 0} \right)}}{{k_{b}^{ - 1} \times \lambda_{b} + \lambda_{p} }}$$

where the fit parameter $C_{{m,{\text{organ}}}} \left( {t = 0} \right)$ represents the initial fraction of injected activity present in the considered animal source organ, while *λ*_b_ and *λ*_p_ are the biologic and the physic decay constants of the mono-exponential fit of the animal data, respectively. This formalism cannot be applied to nTACs of source organs which cannot be fitted with a monoexponential function. In these cases, which in our study concerned only few organs in the [^111^In]CHX-DTPA-scFv78-Fc biodistribution experiment, source organ TIACs were applied unchanged as in M1.

Method 4 (M4): Extrapolation of TIAC_h_ by the combination of the metabolic time scaling used in M3 and the mass scaling used in M2. Therefore, [19, 23]:

4 $${\text{TIAC}}_{h,M4} \left( {{\text{organ}}} \right) = {\text{TIAC}}_{h,M3} \left( {{\text{organ}}} \right) \times \left( {\frac{{m\left( {{\text{organ}}} \right)_{h} /{\text{WB}}_{h} }}{{m\left( {{\text{organ}}} \right)_{m} /{\text{WB}}_{m} }}} \right)$$

For source organs whose nTACs could not be fitted with a monoexponential function, we applied TIACs calculated with M2.

Method 5 (M5): This is an organ-specific implementation of Eq. 3b, where TIAC_h_ are obtained by the application of an allometric scaling according to the equation:

5 $${\text{TIAC}}_{h,M5} \left( {{\text{organ}}} \right) = {\text{TIAC}}_{m} \left( {{\text{organ}}} \right) \times \left( {\frac{{{\text{WB}}_{h} }}{{{\text{WB}}_{m} }}} \right)^{{\beta \left( {{\text{organ}}} \right)}}$$

where the parameter *β* = *b*-1 has been assigned only to selected source organs, namely the liver, the kidneys and the lungs [23]. The corresponding organ-specific b values (i.e. 0.92 for the liver, 0.85 for the kidneys and 1 for the lungs) were reported in Table 2 (all species equations) of [16]. Given the unavailability, in the literature, of β values for source organs other than the liver, the kidneys and the lungs, the TIAC_h_ (*rest of the body*) was obtained by subtraction of TIAC_h_ (*liver*), TIAC_h_ (*lungs*) and TIAC_h_ (*kidneys*), calculated with M5, from the sum of all TIAC_h_ calculated with M1.

**Table 2:** Extrapolated human dosimetry for [^68^Ga]NODAGA-RGDyK

^68^Ga-NODAGA-RGDyK	Average human AD extrapolations (mGy/MBq) (GA subject)				
Target organ	M1	M2	M3	M4	M5
Adrenals	7.40E−03	4.62E−03	1.48E−02	1.12E−02	5.88E−03
Brain	1.68E−03	1.87E−03	6.11E−03	7.36E−03	3.75E−03
Breasts	2.09E−03	2.16E−03	6.78E−03	7.95E−03	4.09E−03
Oesophagus	2.85E−03	2.60E−03	7.67E−03	8.29E−03	4.29E−03
Eyes	1.69E−03	1.88E−03	6.11E−03	7.36E−03	3.75E−03
Gallbladder wall	5.40E−03	3.81E−03	1.12E−02	9.91E−03	5.50E−03
Left colon^†^	1.24E−02	4.01E−03	3.01E−02	1.18E−02	4.49E−03
Small Intestine^†^	2.42E−02	7.20E−03	4.53E−02	1.64E−02	4.43E−03
Stomach Wall^†^	9.20E−03	3.54E−03	2.17E−02	1.05E−02	4.52E−03
Right colon^†^	1.27E−02	4.06E−03	3.06E−02	1.19E−02	4.65E−03
Rectum^†^	1.56E−02	7.63E−03	3.31E−02	1.54E−02	4.37E−03
Heart wall^†^	4.37E−03	3.71E−03	1.08E−02	1.04E−02	4.55E−03
Kidneys^†^	2.71E−02	8.93E−03	5.86E−02	1.96E−02	9.42E−03
Liver^†^	2.81E−02	1.59E−02	3.21E−02	1.87E−02	1.56E−02
Lungs^†^	4.13E−03	6.01E−03	8.79E−03	1.35E−02	4.15E−03
Ovaries	5.38E−03	5.11E−03	1.15E−02	1.21E−02	4.84E−03
Pancreas	4.44E−03	3.19E−03	1.05E−02	9.49E−03	5.01E−03
Prostate	5.86E−03	5.75E−03	1.08E−02	1.14E−02	3.93E−03
Salivary glands	1.79E−03	1.99E−03	6.45E−03	7.77E−03	3.95E−03
Red Marrow^†^	4.21E−03	3.64E−03	1.19E−02	1.11E−02	3.42E−03
Osteogenic cells	2.82E−03	2.54E−03	8.41E−03	8.29E−03	2.96E−03
Spleen^†^	1.68E−02	9.87E−03	2.30E−02	1.41E−02	4.41E−03
Testes	2.48E−03	2.64E−03	6.69E−03	7.82E−03	3.54E−03
Thymus	2.31E−03	2.47E−03	7.26E−03	8.55E−03	4.23E−03
Thyroid	1.94E−03	2.13E−03	6.61E−03	7.94E−03	3.99E−03
Urinary bladder Wall^†^	1.59E−01	1.59E−01	1.64E−01	1.64E−01	4.12E−03
Uterus	9.09E−03	8.61E−03	1.54E−02	1.55E−02	4.82E−03
Total body	4.39E−03	3.80E−03	9.83E−03	9.83E−03	4.39E−03
Effective dose (mSv/MBq)	1.32E−02	1.06E−02	2.09E−02	1.66E−02	4.25E−03

Mouse-to-human dose extrapolations were based on time-integrated activity coefficients (TIACs) obtained from biodistribution data on mice, reported in [25]. Human TIACs were calculated for male and female subjects with five different computational methods and used as input in OLINDA/EXM v.2.1 to derive gender average (GA) organ absorbed doses (ADs) and effective doses (ED) according to the ICRP-103 [32]. ^†^Indicates source organs

TIAC_h_ calculated with the five different methods described above for male and female subjects (Additional file 1: Tables S1 and S2) were used as input in OLINDA/EXM v.2.1 to derive gender average organ AD and effective doses (ED) according to the ICRP-103 [32].

Human dosimetry extrapolated with M2, M3, M4 and M5 was compared with the dosimetry obtained with M1, the most straightforward method for dose extrapolations, using the ratio: M#/M1, where M# = M2, M3, M4, M5.

### [^68^Ga]NODAGA-RGDyK: comparison between mouse-to-human dose extrapolations and human doses

Mouse-to-human dose extrapolations of [^68^Ga]NODAGA-RGDyK were compared with the dosimetry obtained in five male patients with carotid atherosclerosis in a previous clinical study from our group [26]. The patients underwent three whole-body positron emission tomography/computed tomography (PET/CT) scans (Discovery 690, GE Healthcare, Milwaukee, Wisconsin, USA) 10, 60 and 120 min after the intravenous radiopharmaceutical injection. The following body regions were manually segmented on the CT part of every PET/CT study: brain, thyroid, lungs, heart, liver, spleen, stomach, kidneys, red marrow, pancreas, small intestine, colon and whole body. Choroid plexuses and urinary bladder were segmented on the emission PET data. Given the short half-life of ^68^Ga, all organ TIACs could be obtained by fitting nTACs with a monoexponential function extended to infinite beyond the last measured data point. Full details on the PET/CT acquisition, reconstructions and analysis protocols including organ segmentation were reported elsewhere [26].

For the purpose of the present study, source organ TIACs obtained in patients were used as input in OLINDA/EXM v.2.1. Organ ADs, either obtained directly in male patients or extrapolated to female subjects, were used to calculate organ ADs and ED of the gender average subject according to the ICRP103 methodology [32]. The choroid plexuses, which showed an intense [^68^Ga]NODAGA-RGDyK uptake, do not appear in the list of available organs in OLINDA and were the object of a specific dosimetry modelling [33]. [^68^Ga]NODAGA-RGDyK human dosimetry extrapolated from mice with M1, M2, M3, M4 and M5 was compared with the dosimetry obtained in human subjects using the ratio M#/H, where M# = M1, M2, M3, M4, M5.

We used organ-specific biologic half-life *τ*_b,h_ and *τ*_b,m_, from [25] and [26], respectively, to evaluate organ-specific *k*_b_ from Eq. 3a. Subsequently, we estimated organ-specific α′ values by solving equation Eq. 3b:

6 $$\alpha^{{\prime }} = \log_{{\left( {\frac{{WB_{h} }}{{WB_{m} }}} \right)}} k_{b}$$

Finally, we estimated a [^68^Ga]NODAGA-RGDyK-specific α_new_ value from the average of the calculated source organ α′ values.

Lastly, we calculated organ-specific *β*′ values by solving Eq. 5 for *β*:

7 $$\beta^{\prime } \left( {{\text{organ}}} \right) = \log_{{\frac{{{\text{WB}}_{h} }}{{{\text{WB}}_{m} }}}} \left( {\frac{{{\text{TIAC}}_{h} \left( {{\text{organ}}} \right)}}{{{\text{TIAC}}_{m} \left( {{\text{organ}}} \right)}}} \right)$$

where TIAC_h_ are taken from [26], and TIAC_m_ are taken from [25].

The uncertainties on biological decay times *τ*_b_ were estimated with the standard deviations $\sigma_{{\tau_{b,h} }}$ and $\sigma_{{\tau_{b,m} }}$ obtained from the clinical and the preclinical studies, respectively. The uncertainty on *k*_b_ was estimated by the error propagation as follows:

8 $${\Delta }k_{b} = \sqrt {\left( {\frac{{\sigma_{{\tau_{b,h} }} }}{{\tau_{b,m} }}} \right)^{2} + \left( {\frac{{\tau_{b,h} }}{{\tau_{b,m}^{2} }}} \right)^{2} \sigma_{{\tau_{b,m} }}^{2} }$$

The error propagation was applied to obtain estimates of the uncertainties of *α*' and *β*′ using the following equations:

9 $$\Delta \alpha^{\prime } = \left| {\frac{{\Delta k_{b} }}{{k_{b} \times \ln \left( {\frac{{WB_{h} }}{{WB_{m} }}} \right)}}} \right|$$

10 $$\Delta \beta^{\prime } = \left| {\frac{{\Delta \left( {\frac{{TIAC_{h} }}{{TIAC_{m} }}} \right)}}{{\left( {\frac{{TIAC_{h} }}{{TIAC_{m} }}} \right) \times \ln \left( {\frac{{WB_{h} }}{{WB_{m} }}} \right)}}} \right|$$

with

11 $$\Delta \left( {\frac{{{\text{TIAC}}_{h} }}{{{\text{TIAC}}_{m} }}} \right) = \sqrt {\left( {\frac{{\sigma_{{{\text{TIAC}}_{h} }} }}{{{\text{TIAC}}_{m} }}} \right)^{2} + \left( {\frac{{{\text{TIAC}}_{h} }}{{{\text{TIAC}}_{m}^{2} }}} \right)^{2} \times \sigma_{{{\text{TIAC}}_{m} }}^{2} }$$

where $\sigma_{{TIAC_{h} }}$ and $\sigma_{{TIAC_{m} }}$ for each considered organ were derived from the clinical and preclinical studies, respectively. In order to investigate the validity of this experimentally driven allometric approach, the α_new_ value was applied to Eqs. 3b, 3c and 4 to extrapolate an additional set of TIAC_h_ and the corresponding source organ ADs, using M3 and M4, respectively.

### Statistical analysis

The results obtained with the different methods for dose extrapolations were compared with the Friedman test for multiple comparisons of paired data. Multiple head-to-head comparisons between dose extrapolations were assessed with the Wilcoxon matched-pairs signed-rank test (GraphPad Prism Version 8, GraphPad Software, San Diego, CA). Probability values of less than 0.05 were considered significant.

## Results

### Human dose extrapolations from mouse data

Extrapolated human dosimetry of [^111^In]CHX-DTPA-scFv78-Fc and [^68^Ga]NODAGA-RGDyK is reported in Tables 1 and 2, respectively. For both radiopharmaceuticals, human organ ADs obtained with the five extrapolation methods were significantly different (both *p* < 0.0001, Friedman test). These figures did not change when only ADs to source organs for which TIAC_m_ were directly calculated from biodistribution studies were considered for comparisons (all *p* < 0.0001, Friedman test).

Head-to-head comparisons between methods for dose extrapolations provided statistically significant differences in most cases (full data not shown). Interestingly, for both radiopharmaceuticals, organ ADs obtained with the most widely used methods for dose extrapolations M1 and M2 were significantly different. This was confirmed considering either all organs (*p* < 0.0001 and *p* = 0.0005 for [^111^In]CHX-DTPA-scFv78-Fc and [^68^Ga]NODAGA-RGDyK, respectively, Wilcoxon signed-rank test), or only source organs for which TIAC_m_ were directly calculated from biodistribution studies (*p* = 0.0012 and *p* = 0.0034 for [^111^In]CHX-DTPA-scFv78-Fc and [^68^Ga]NODAGA-RGDyK, respectively, Wilcoxon signed-rank test). In general, organ ADs obtained with M3 were significantly higher than those obtained with the other methods for dose extrapolations (all *p* < 0.005, Wilcoxon signed-rank test). An exception to this was the non-significant difference between M3 and M4 for [^68^Ga]NODAGA-RGDyK (*p* = 0.17, Wilcoxon signed-rank test) although, if only source organs were considered, statistical significance was retained (*p* = 0.0049, Wilcoxon signed-rank test). Also, ADs obtained with M5 were similar to those obtained with M2 (*p* = 0.83 and *p* = 0.48 for [^111^In]CHX-DTPA-scFv78-Fc and [^68^Ga]NODAGA-RGDyK, respectively, Wilcoxon signed-rank test).

Extrapolated organ AD ratios between M2, M3, M4, M5 and M1 for [^111^In]CHX-DTPA-scFv78-Fc and [^68^Ga]NODAGA-RGDyK are reported in Additional file 1: Tables S3 and S4, respectively.

### [^68^Ga]NODAGA-RGDyK: mouse-to-human dose extrapolations vs. human doses

M1, M2, M4 and M5 for mouse-to-human dose extrapolations resulted in ADs significantly different from those calculated directly on human subjects (all* p* ≤ 0.001, Wilcoxon signed-rank tests)*.* In contrast, no significant differences were found between ADs calculated with M3 and those obtained directly on humans (*p* = 0.99 considering all organs; *p* = 0.339 considering only source organs, Wilcoxon signed-rank tests). Human organ ADs and ED, as well as organ AD ratios, are reported in Table 3.

**Table 3:** Comparison between mice-to-human dose extrapolations and dosimetry directly calculated on human subjects for [^68^Ga]NODAGA-RGDyK

^68^Ga-NODAGA-RGDyK		AD ratios				
Target Organ	Human organ AD (mGy/MBq)	M1/H	M2/H	M3/H	M4/H	M5/H
Adrenals	1.55E−02	0.48	0.30	0.95	0.72	0.38
Brain	2.34E−03	0.72	0.80	2.61	3.15	1.60
Breasts	8.93E−03	0.23	0.24	0.76	0.89	0.46
Oesophagus	9.52E−03	0.30	0.27	0.81	0.87	0.45
Eyes	7.84E−03	0.22	0.24	0.78	0.94	0.48
Gallbladder Wall	1.17E−02	0.46	0.33	0.96	0.85	0.47
Left colon	2.57E−02	0.48	0.16	1.17	0.46	0.17
Small Intestine	4.37E−02	0.55	0.16	1.04	0.38	0.10
Stomach Wall	1.64E−02	0.56	0.22	1.32	0.64	0.28
Right colon	2.57E−02	0.49	0.16	1.19	0.46	0.18
Rectum	2.73E−02	0.57	0.28	1.21	0.56	0.16
Heart Wall	1.78E−02	0.25	0.21	0.61	0.58	0.26
Kidneys	4.74E−02	0.57	0.19	1.24	0.41	0.20
Liver	1.92E−02	1.46	0.83	1.67	0.97	0.81
Lungs	1.49E−02	0.28	0.40	0.59	0.91	0.28
Ovaries	1.28E−02	0.42	0.40	0.90	0.95	0.38
Pancreas	1.03E−02	0.43	0.31	1.02	0.92	0.49
Prostate	1.12E−02	0.52	0.51	0.96	1.02	0.35
Salivary Glands	8.36E−03	0.21	0.24	0.77	0.93	0.47
Red Marrow	1.06E−02	0.40	0.34	1.12	1.05	0.32
Osteogenic Cells	8.20E−03	0.34	0.31	1.03	1.01	0.36
Spleen	3.80E−02	0.44	0.26	0.61	0.37	0.12
Testes	8.24E−03	0.30	0.32	0.81	0.95	0.43
Thymus	9.72E−03	0.24	0.25	0.75	0.88	0.44
Thyroid	1.11E−02	0.17	0.19	0.60	0.72	0.36
Urinary Bladder Wall	1.04E−01	1.53	1.53	1.58	1.58	0.04
Uterus	1.52E−02	0.60	0.57	1.01	1.02	0.32
Total Body	1.10E−02	0.40	0.35	0.89	0.89	0.40
Average organ AD ratio	–	0.49	0.37	1.03	0.90	0.38
ED (mSv/MBq)	1.78E−02	0.74	0.60	1.17	0.93	0.24

Source organ time-integrated activity coefficients obtained in five male patients were taken from [26], and used as input in OLINDA/EXM v.2.1 to derive absorbed doses (ADs) and effective doses (ED) to the gender average (GA) subject. AD ratios were calculated to compare different methods for mice-to-human extrapolations (M1-5) with ADs calculated on human subjects (H)

**Fig. 1:**
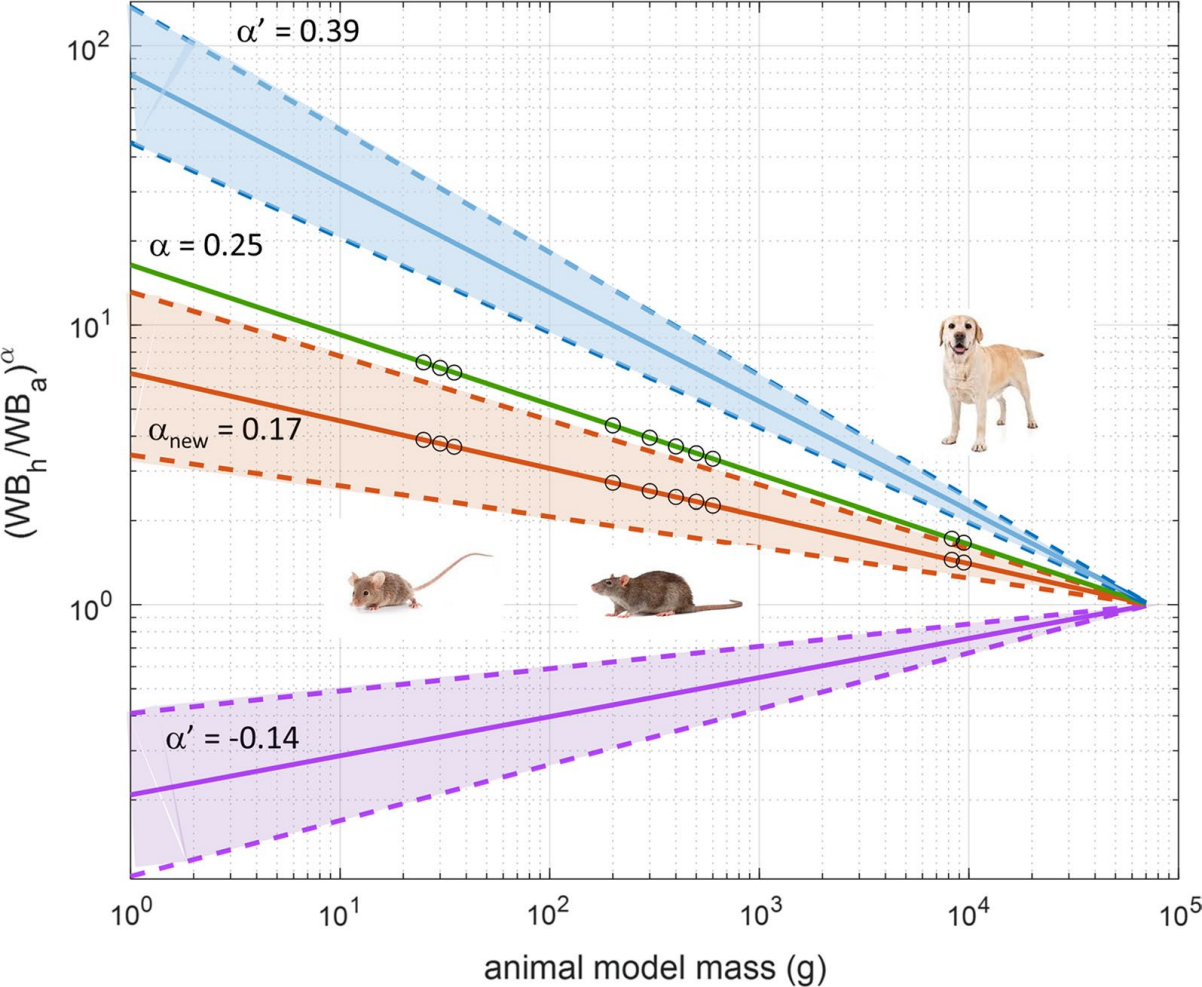
Variation of the scaling factor *k*_*b*_ = (WB_h_/WB_a_)^*α*^ as a function of the animal body mass. The figure shows how the scaling factor (WB_h_/WB_a_)^*α*^, applied in M3 and M4, varies according to the animal body mass, for a mass of 73 kg (human adult male reference phantom). Four curves are presented, considering the reference α = 0.25 (green line) [19, 20, 23], the *α*_new_ = 0.17 (orange line) obtained experimentally for [^68^Ga]NODAGA-RGDyK, as well as the lowest and the highest organ *α*′ values, i.e. *α*′ = − 0.14 (magenta line) and *α*′ = 0.39 (blue line), found in this study for liver and red marrow, respectively. The shadow areas represent the uncertainties around the calculation of α_new_ and of α’ extremes (see Table 4). The circles represent the scaling factors corresponding to the masses of the murine, rat and canine models implemented in OLINDA/EXM 2.0. In the case of a 25 g mouse, the scaling factors are 7.35 and 3.88 for *α* = 0.25 and *α* = 0.17, respectively. Images of animals and humans are copyright-free images taken from the Internet (dreamstime.com)

Organ-specific *α*′ and *β*', calculated using combined experimental biodistribution data from mice and humans, are reported in Table 4, along with their respective average values *α*_new_ = 0.17 and *β*_mean_ = 0.08. The organ ADs obtained with M3 and M4 by the application of *α*_new_ = 0.17 are reported in Table 5. Newly calculated ADs were significantly different from those previously obtained by the application of α = 0.25 (both *p* < 0.001, Wilcoxon signed-rank tests). Considering only the source organs, the application of *α*_new_ = 0.17 leads to a change from 1.07 to 0.94 of the average M3/H, and from 0.62 to 0.54 of the average M4/H (Additional file 1: Table S5). Figure 1 shows the variation of the metabolic scaling factor *k*_*b*__=_ (WB_h_/WB_a_)^*α*^ presented in Eqs. 3 and 4 as a function of the animal body mass if different *α* values are applied.

**Table 4:** Organ-specific allometric parameters, and corresponding uncertainties, calculated using combined experimental [^68^Ga]NODAGA-RGDyK biodistribution data from mice and humans

Organ	*τ*_b,m_ (h)	*τ*_b,m_	*τ*_b,h_ (h)	*στ*_b,h_	*k*_*b*_	Δ*k*_*b*_	*α*'	Δ*α*'	*β*′	Δ*β*′
Liver	15.12	4.54	4.91	1.84	0.32	0.16	− 0.14	0.06	− 0.06	0.05
Kidneys	0.69	0.21	2.38	0.66	3.42	1.39	0.16	0.05	0.07	0.08
Lung	0.69	0.21	1.94	0.10	2.79	0.85	0.13	0.04	0.19	0.04
Spleen	4.21	1.26	17.59	0.11	4.17	1.25	0.18	0.04	0.10	0.09
Heart wall	0.47	0.14	1.28	0.22	2.74	0.95	0.13	0.04	0.26	0.05
Stomach	0.69	0.21	4.29	5.45	6.17	8.06	0.23	0.16	0.00	0.06
Small intestine	1.19	0.36	4.07	1.90	3.14	1.89	0.16	0.07	0.06	0.05
Left Colon	0.60	0.18	3.50	1.40	5.87	2.94	0.22	0.06	0.06	0.05
Right colon	0.60	0.18	3.50	1.40	5.87	2.94	0.22	0.06	0.06	0.05
Rectum	0.60	0.18	3.50	1.40	5.87	2.94	0.22	0.06	0.06	0.05
Red marrow	0.53	0.16	11.65	3.58	22.4	9.46	0.39	0.05	0.07	0.04
Average of all values							0.17 (α_new_)	0.06	0.08 (β_mean_)	0.05

**Table 5:** [^68^Ga]NODAGA-RGDyK: M3 and M4 reassessed using α_new_ = 0.17

Target Organ	Organ ADs (mGy/MBq) GA subject	
	M3	M4
Adrenals	1.30E−02	9.70E−03
Brain	4.96E−03	6.10E−03
Breasts	5.56E−03	6.62E−03
Oesophagus	6.43E−03	7.02E−03
Eyes	4.96E−03	6.10E−03
Gallbladder Wall	9.73E−03	8.52E−03
Left colon	2.55E−02	1.00E−02
Small Intestine	4.07E−02	1.43E−02
Stomach Wall	1.85E−02	8.92E−03
Right colon	2.60E−02	1.00E−02
Rectum	2.86E−02	1.36E−02
Heart Wall	9.02E−03	8.15E−03
Kidneys	5.10E−02	1.70E−02
Liver	3.14E−02	1.82E−02
Lungs	7.64E−03	1.17E−02
Ovaries	9.90E−03	1.05E−02
Pancreas	8.97E−03	8.05E−03
Prostate	9.52E−03	1.01E−02
Salivary Glands	5.24E−03	6.44E−03
Red Marrow	9.96E−03	9.34E−03
Osteogenic Cells	7.00E−03	6.96E−03
Spleen	2.19E−02	1.33E−02
Testes	5.60E−03	6.64E−03
Thymus	5.97E−03	7.19E−03
Thyroid	5.40E−03	6.60E−03
Urinary Bladder Wall	1.62E−01	1.63E−01
Uterus	1.37E−02	1.39E−02
Total Body	8.45E−03	8.45E−03
ED (mSv/MBq)	1.90E−02	1.52E−02

## Discussion

Recently, internal dosimetry has greatly expanded, because of the accumulation of clinical evidence of dose/response correlations for a number of radiopharmaceuticals [34–37], and of the improved hardware and software technology, which allows more accurate and reproducible results across centres. Dosimetry assessed on laboratory animals has the recognized potential to anticipate the toxicity and efficacy profiles of new radiopharmaceuticals. Nevertheless, it presents several challenges and lacks standardization [9, 38, 39]. In general, bigger mammals are considered better candidates than rodents for the extrapolation of radiopharmaceutical biodistribution in humans. However, most of the laboratory experiments in modern onco-immunology are currently being conducted on small rodents. Mice are considered the mainstay, because they present several practical advantages over rats in terms of cost, easiness of breeding and of genetical manipulation [9]. In an effort to contribute to the standardization of translational dosimetry, in this article we focused on the computational aspects of mouse-to-human AD extrapolations.

Firstly, we compared five available methods for mouse-to-human AD extrapolations on two different radiopharmaceuticals. The choice was primarily based on the fact that we had access to full raw experimental data in small animals, as both radiopharmaceuticals were tested preclinically in our centre. One of these radiotracers, the [^68^Ga]NODAGA-RGDyK, is currently being used in human patients [26–29], and published human dosimetry data are available for comparison with AD extrapolations [26]. Furthermore, the diversity of these two radiotracers may allow to generalize our findings to targeted macromolecules with various physical and biological characteristics. The two radiopharmaceuticals under study are based on macromolecules of different size, conjugated with two different bifunctional chelators. The [^111^In]CHX-DTPA-scFv78-Fc is based on a 120 kDa fusion protein [40], whereas [^68^Ga]NODAGA-RGDyK is based on three amino acids, namely arginine–glycine–aspartic acid (RGD), forming a small cyclic peptide [41]. Physical half-lives of ^111^In and ^68^Ga are also considerably different: 67 h vs. 68 min, respectively. In the five equations, allometric scaling is applied with increasing levels of complexity, ranging from the simple direct application of TIACs_m_ to human organs (i.e. M1) to more refined equations including organ-specific metabolic time and mass scaling.

We showed that average ADs calculated with the two most popular methods for dose extrapolations, that are the direct extrapolation method and the method applying a relative mass scaling factor (here referred as to M1 and M2), were significantly different. On average, doses obtained with M2 were significantly lower than those calculated with M1, for both [^111^In]CHX-DTPA-scFv78-Fc and [^68^Ga]NODAGA-RGDyK. This is coherent with the fact that, in mice, most of the organ masses are relatively larger than in humans, compared to their respective total body masses. These findings are in contrast with the results of Maina et al., who showed no statistically significant differences of average ADs between M1 and M2 for dose extrapolations of the cholecystokinin analogue [^111^In]-CP04 [22]. This could be attributed to differences between the radiopharmaceuticals used in the two studies, or to the methodology adopted for the calculation of some specific source organ TIACs, such as the hollow organs or the blood. However, the results of Maina et al. for single, relevant organs such as the kidneys, the liver and the spleen were in line with our findings, with the direct extrapolation method providing higher ADs than the method adopting the relative mass scaling factor [22].

The other methods for dose extrapolations (M3, M4 and M5) introduce additional scaling components aiming to correct for differences of metabolic rates across species. M3 and M4 apply the same correction factor, *k*_*b*_, that scales the biological component of the effective half-life (see Eq. 3b). This correction factor depends on the ratio between the human and the specific animal mass used for the preclinical experiment, and on the power constant α = 0.25, which is applied unmodified to all organs. The power constant α = 0.25 has been derived empirically to match the average heart and respiratory rates across most common mammal models [16, 19]. In contrast, in M5, the relative mass scaling, applied to the animal TIAC, considers a power constant factor β that is organ-specific (see Eq. 4). Compared to M1, the organ-specific factor β, available for three organs only (i.e. the liver, the kidneys and the lungs [16]), assumes values ≤ 0, which has the net effect to reduce the corresponding organ TIAC, whereas the metabolic scaling proposed in M3 and M4 goes in the opposite direction of incrementing the organ TIACs. Owing to the positivity of the α value, which results in an increase in the biological radiopharmaceutical half-life in humans compared to laboratory animals, in our study the dose extrapolations calculated with M3 and M4 were higher than those obtained with the other methods. ADs obtained with M4 were, on average, lower than those obtained with M3. This is due to the fact that M4 includes the relative mass scaling factor, whereas M3 does not, as discussed above regarding the differences between M1 and M2. Another result that deserves to be discussed is the increment of ADs calculated with M3 and M4, relative to M1, for the two different radiopharmaceuticals. In the case of [^68^Ga]NODAGA-RGDyK, M3/M1 and M4/M1 ratios were much higher than those obtained for [^111^In]CHX-DTPA-scFv78-Fc. This can be explained by the interplay between the biological and the physical radiopharmaceutical half-lives in Eq. 3c. In fact, according to Eq. 3c, for radiopharmaceuticals with similar biological and physical half-lives, the extrapolated source organ TIAC_h_ is lower than the source organ TIAC_h_ obtained for radiopharmaceuticals characterized by a significantly larger biological half-life compared to the physical half-life.

The second objective of our study was to compare AD extrapolations with the ADs directly calculated on human subjects. This was possible only for the [^68^Ga]NODAGA-RGDyK, for which human dosimetry data were available. Conflicting results exist in the literature regarding the accuracy of animal-to-human AD extrapolations, and none of the available computational methods showed a clear superiority over the others. Although there are guidelines, like the Swiss federal guidelines [42], recommending M2 for dosimetry extrapolations, this field remains largely unexplored. Irrespective of the methods and of the animal model adopted, differences between animal-to-human extrapolations and experimentally obtained dosimetry in human subjects were in the range 20–50% at best [43–45]. In our hands, the dose extrapolations obtained with M3 were the most similar to the ADs directly calculated on human subjects (i.e. non-significant differences, *p* = 0.99), whereas ADs calculated with the other methods were significantly different from the human benchmark. This is in accordance with the results of Beykan et al., who showed that the extrapolations obtained with M3 and M4 from mice data were in better agreement with the human dosimetry of the somatostatin receptor antagonist [^177^Lu]-OPS201 than those obtained with other methods [23].

Lastly, we retrospectively compared the available [^68^Ga]NODAGA-RGDyK experimental mice and human biological half-lives to extrapolate organ-specific metabolic scaling factors *k*_*b*_ and, upon solution of Eq. 3b, the corresponding *α*′ values. The *α*_new_ value was obtained from the average of the source organ *α*′ values. The application of *α*_new_ = 0.17 to M3 and M4 resulted in significantly different organ ADs compared with the application of *α* = 0.25. However, in terms of average ADs for most relevant source organs, the application of *α*_new_ = 0.17 to M3 and M4 resulted in only small percent differences compared to the application of *α* = 0.25. Notably, the metabolic time scaling showed a large heterogeneity within the different source organs, with *α*′ assuming both negative and positive values. These ranged from *α*′ = − 0.14 (*τ*_b_ in human < *τ*_b_ in the animal model) for the liver to *α*′ = 0.39 for the red marrow. It appears, therefore, that the adoption of the same metabolic rate scaling for a given animal model does not correctly represent the radiopharmaceutical metabolism of single organs, possible leading to large errors in human AD extrapolations.

Figure 1 shows how the metabolic rate scaling *k*_*b*_ changes across different animal models by the adoption of *α* = 0.25, *α*_new_ = 0.17, as well as in case of the two extreme *α*′ values found in the present work, i.e. *α*′ = − 0.14 (liver) and *α*′ = 0.39 (red marrow), respectively.

A limitation of the present study is that, for simplicity, we only considered average animal and human AD values without taking into account the uncertainty associated with either estimation. The degree of uncertainty associated with the dosimetry data on which the present extrapolations are based was, on average, about 20–25% in both human and mouse experiments [24–26]. This means that, at least for the [^68^Ga]NODAGA-RGDyK, M3 and M4 would likely result to be superior to the other methods for AD extrapolations in predicting human ADs even if the associated uncertainties were taken into account. Nevertheless, the intrinsic biological heterogeneity of the animal models, although generally smaller than the heterogeneity observed among humans, as well as the intrinsic errors associated with AD calculations, should always be considered in translational radiopharmaceutical research. Furthermore, the possible influence of gender differences on dose extrapolations and human dose predictions could not be assessed by the present analysis, as all animal experiments were performed in female mice, whereas the human [^68^Ga]NODAGA-RGDyK study was performed in male subjects [24–26]. Another limitation is represented by the number of available time-points in both preclinical and clinical experiments. The limited number of time points restricted our choice to the use of a mono-exponential fit for source organ TACs to derive biological half-lives (*τ*_b_) in the formalisms of M3 and M4. If more data points were available, organ TIACs could have been obtained including multiple terms as described in Eq. 14 of [19]. An additional comment that should be made on our results concerns their generalizability. Our considerations regarding methods for AD extrapolations were based on two radiopharmaceuticals with significantly different biological and physical characteristics. The comparison with human dosimetry data regarded only one of these two radiopharmaceuticals. A larger generalization of our results would require the extension of our methodological approach to more radiopharmaceuticals and to preclinical models other than mice.

## Conclusions

Radiation dose estimates based on animal data are a necessary part of the drug approval process. However, they are of limited value due to significant variability of human dose predictions, depending on the computational method adopted for absorbed dose extrapolation. Here, we discuss the methodological implications of the different available methods for mouse-to-human dosimetry extrapolations, demonstrating that they provide significantly different results on two targeted radiopharmaceuticals. For the α_v_β_3_ integrin-targeting [^68^Ga]NODAGA-RGDyK, we showed that the best approximation of the actual human dosimetry was provided by the extrapolation method applying a metabolic scaling to the mouse organ TIACs (i.e. M3). The formulation of more refined extrapolation algorithms applying various combinations of organ metabolic and mass scaling requires further investigation, as their accuracy might be improved by the determination of appropriate, model-specific metabolic scaling parameters.

## Supplementary Information

**Additional file 1**. Tables S1-S5. 

## Data Availability

The data sets used and/or analysed during the current study are available from the corresponding author on reasonable request.

## Abbreviations

AD: Absorbed dose
TIACs: Time-integrated activity coefficients
DTPA: Diethylenetriaminepentaacetic acid
NODAGA: 1,4,7-Triazacyclononane,1-glutaric acid-4,7-acetic acid
RGD: Arginine–glycine–aspartic acid
scFv: Single-chain variable fragment
TEM-1: Tumour endothelial marker 1
Fc: Crystallizable fragment
nA: Normalized injected activity
nTACs: Normalized time–activity curves
WB: Whole body
ICRP: International Commission of Radiation Protection
ED: Effective doses
PET/CT: Positron emission tomography/computed tomography
