# Supplementary material for: Comparison of absorbed dose extrapolation methods for mouse-to-human translation of radiolabelled macromolecules
Source: EJNMMI Res. 2022 Apr 11;12:21. doi: 10.1186/s13550-022-00893-z (PMC9001797; doi:10.1186/s13550-022-00893-z)
Supplement: Supplementary file 1 — Additional file 1. Tables S1-S5. [file 13550_2022_893_MOESM1_ESM.docx]

**Supplementary Table 1.** Source organ TIACs for the [^68^Ga]NODAGA-RGDyK.

|  | Buchegger et al. [25] | |  | | | | | | | | |
| --- | --- | --- | --- | --- | --- | --- | --- | --- | --- | --- | --- |
|  | M1 | | M2 | | M3 | | M4 | | M5 | | Gnesin et al. [26] |
| Source Organ | M | F | M | F | M | F | M | F | M | F | M |
| Brain |  |  |  |  |  |  |  |  |  |  | 3.20E-02 |
| Thyroid |  |  |  |  |  |  |  |  |  |  | 3.99E-04 |
| Liver | 8.53E-02 | 8.53E-02 | 4.91E-02 | 4.65E-02 | 9.28E-02 | 9.27E-02 | 5.35E-02 | 5.06E-02 | 4.54E-02 | 4.62E-02 | 5.11E-02 |
| Kidneys | 1.64E-02 | 1.64E-02 | 4.72E-03 | 5.12E-03 | 3.62E-02 | 3.58E-02 | 1.04E-02 | 1.12E-02 | 5.03E-03 | 5.18E-03 | 2.86E-02 |
| Lung | 6.80E-03 | 6.80E-03 | 1.29E-02 | 1.25E-02 | 1.49E-02 | 1.48E-02 | 2.84E-02 | 2.71E-02 | 6.80E-03 | 6.80E-03 | 3.01E-02 |
| Spleen | 4.80E-03 | 4.80E-03 | 2.74E-03 | 2.89E-03 | 6.03E-03 | 6.01E-03 | 3.44E-03 | 3.62E-03 |  |  | 1.08E-02 |
| Heart wall | 1.80E-03 | 1.80E-03 | 1.62E-03 | 1.49E-03 | 4.75E-03 | 4.69E-03 | 4.27E-03 | 3.88E-03 |  |  | 1.41E-02 |
| Stomach | 6.10E-03 | 6.10E-03 | 8.50E-04 | 9.65E-04 | 1.34E-02 | 1.33E-02 | 1.86E-03 | 2.10E-03 |  |  | 6.16E-03 |
| Small intestine | 2.81E-02 | 2.81E-02 | 5.19E-03 | 5.83E-03 | 4.95E-02 | 4.92E-02 | 9.15E-03 | 1.02E-02 |  |  | 4.52E-02 |
| Left Colon | 3.08E-03 | 3.08E-03 | 3.74E-04 | 4.24E-04 | 7.31E-03 | 7.23E-03 | 8.88E-04 | 9.98E-04 |  |  | 5.12E-03 |
| Right colon | 6.15E-03 | 6.15E-03 | 7.47E-04 | 8.79E-04 | 1.46E-02 | 1.45E-02 | 1.78E-03 | 2.07E-03 |  |  | 1.02E-02 |
| Rectum | 3.08E-03 | 3.08E-03 | 3.74E-04 | 4.24E-04 | 7.31E-03 | 7.23E-03 | 8.88E-04 | 9.98E-04 |  |  | 5.12E-03 |
| Red marrow | 8.30E-03 | 1.01E-02 | 6.37E-03 | 7.25E-03 | 2.82E-02 | 2.79E-02 | 2.16E-02 | 2.00E-02 |  |  | 1.44E-02 |
| Pancreas |  |  |  |  |  |  |  |  |  |  | 1.43E-02 |
| Bladder | 1.20E-01 | 1.20E-01 | 1.20E-01 | 1.20E-01 | 1.20E-01 | 1.20E-01 | 1.20E-01 | 1.20E-01 |  |  | 7.32E-02 |
| Rest of body | 1.80E-01 | 1.78E-01 | 2.03E-01 | 2.00E-01 | 6.62E-01 | 6.53E-01 | 8.00E-01 | 7.94E-01 | 4.13E-01 | 4.13E-01 | 8.77E-01 |

Human source organ time-integrated activity coefficients (TIACs) are calculated from mice data with five different computational methods (M1-M5) for the male (M) and female subjects (F). TIACs calculated with M1 for the female and male subjects were taken from Table 1 of [25]. Human organ TIACs calculated on five male patients were taken from Table 2 of [26].

**Supplementary Table 2.** Source organ TIACs for the [^111^In]CHX-DTPA-scFv78-Fc.

|  | M1 | M2 | | M3 | | M4 | | M5 | |
| --- | --- | --- | --- | --- | --- | --- | --- | --- | --- |
| Source organ | F | M | F | M | F | M | F | M | F |
| Blood (total) | 2.94E+00 | 3.38E+00 | 2.99E+00 | 6.07E+00 | 5.91E+00 | 6.98E+00 | 6.80E+00 |  |  |
| Red marrow | / | 3.51E-01 | 3.10E-01 | 2.73E+00 | 2.66E+00 | 2.73E+00 | 2.66E+00 |  |  |
| Heart cavity | / | 8.06E-01 | 7.54E-01 | 1.16E+00 | 1.13E+00 | 1.16E+00 | 1.13E+00 |  |  |
| Liver | 2.11E+01 | 1.34E+01 | 1.27E+01 | 1.95E+01 | 1.95E+01 | 1.24E+01 | 1.24E+01 | 1.11E+01 | 1.13E+01 |
| Spleen | 2.08E+00 | 2.74E+00 | 2.88E+00 | 2.08E+00 | 2.08E+00 | 2.74E+00 | 2.74E+00 |  |  |
| Heart wall | 1.59E-01 | 1.81E-01 | 1.67E-01 | 5.89E-01 | 5.77E-01 | 6.71E-01 | 6.57E-01 |  |  |
| Kidneys | 2.69E+00 | 1.11E+00 | 1.21E+00 | 5.26E+00 | 5.22E+00 | 2.17E+00 | 2.15E+00 | 8.13E-01 | 8.37E-01 |
| Lungs | 6.97E-01 | 1.66E+00 | 1.60E+00 | 1.76E+00 | 1.74E+00 | 4.19E+00 | 4.14E+00 | 6.97E-01 | 6.97E-01 |
| Uterus | 1.63E+00 |  | 2.45E-01 |  | 1.63E+00 |  | 1.63E+00 |  |  |
| Ovaries | 2.24E-01 |  | 3.37E-02 |  | 2.24E-01 |  | 2.24E-01 |  |  |
| Stomach | 6.65E-01 | 5.82E-02 | 6.61E-02 | 6.65E-01 | 6.65E-01 | 5.82E-02 | 5.82E-02 |  |  |
| Pancreas | 2.04E-01 | 9.49E-02 | 9.90E-02 | 2.15E-01 | 2.15E-01 | 1.00E-01 | 1.00E-01 |  |  |
| Small intestine | 7.47E+00 | 1.32E+00 | 1.48E+00 | 8.35E+00 | 8.35E+00 | 1.48E+00 | 1.47E+00 |  |  |
| Left colon | 5.61E-01 | 7.56E-02 | 8.58E-02 | 1.02E+00 | 1.01E+00 | 1.37E-01 | 1.36E-01 |  |  |
| Right colon | 1.12E+00 | 1.51E-01 | 1.78E-01 | 2.04E+00 | 2.02E+00 | 2.75E-01 | 2.73E-01 |  |  |
| Rectum | 5.61E-01 | 7.56E-02 | 8.58E-02 | 1.02E+00 | 1.01E+00 | 1.37E-01 | 1.36E-01 |  |  |
| Remainder | 1.02E+01 | 1.58E+01 | 1.29E+01 | 1.53E+01 | 1.53E+01 | 2.37E+01 | 2.37E+01 | 3.80E+01 | 3.78E+01 |
| Total TIAC | 4.94E+01 | 3.78E+01 | 3.48E+01 | 6.78E+01 | 6.92E+01 | 5.89E+01 | 6.03E+01 | 5.07E+01 | 5.07E+01 |

Human source organ time-integrated activity coefficients (TIACs) are calculated from female mice data with five different computational methods (M1-M5) for the male (M) and female subjects (F). TIACs for the female subject calculated with M1 were taken from Supplementary Table 2 of [24].

**Supplementary Table 3.** Extrapolated human absorbed dose (AD) and effective dose (ED) ratios for [^111^In]CHX-DTPA-scFv78-Fc.

| **[^111^In]CHX-DTPA-scFv78-Fc** | **Human AD ratios**  **(GA subject)** | | | |
| --- | --- | --- | --- | --- |
| **Target Organ** | **M2/ M1** | **M3/ M1** | **M4/ M1** | **M5/ M1** |
| Adrenals | 0.73 | 1.18 | 0.82 | 0.48 |
| Brain | 1.40 | 1.66 | 2.41 | 3.42 |
| Breasts | 0.82 | 1.23 | 1.34 | 1.35 |
| Esophagus | 0.71 | 1.12 | 0.93 | 0.73 |
| Eyes | 1.39 | 1.66 | 2.39 | 3.38 |
| Gallbladder Wall | 0.59 | 1.09 | 0.65 | 0.58 |
| Left colon | 0.38 | 1.46 | 0.56 | 0.36 |
| Small Intestine | 0.27 | 1.16 | 0.34 | 0.18 |
| Stomach Wall | 0.55 | 1.11 | 0.66 | 0.47 |
| Right colon | 0.37 | 1.48 | 0.50 | 0.39 |
| Rectum | 0.29 | 1.57 | 0.59 | 0.38 |
| Heart Wall | 0.55 | 1.21 | 1.15 | 0.50 |
| Kidneys | 0.52 | 1.67 | 0.79 | 0.39 |
| Liver | 0.62 | 0.96 | 0.62 | 0.54 |
| Lungs | 0.97 | 1.34 | 1.35 | 0.80 |
| Ovaries | 0.19 | 1.05 | 0.98 | 0.13 |
| Pancreas | 0.53 | 1.10 | 0.60 | 0.46 |
| Prostate | 0.69 | 1.46 | 1.00 | 1.23 |
| Salivary Glands | 1.33 | 1.57 | 2.22 | 3.10 |
| Red Marrow | 0.92 | 1.87 | 1.69 | 1.08 |
| Osteogenic Cells | 0.88 | 1.58 | 1.50 | 1.40 |
| Spleen | 1.23 | 1.06 | 1.24 | 0.14 |
| Testes | 1.36 | 1.54 | 2.03 | 3.04 |
| Thymus | 0.80 | 1.24 | 1.32 | 1.04 |
| Thyroid | 1.10 | 1.41 | 1.70 | 1.86 |
| Urinary Bladder Wall | 0.55 | 1.34 | 1.09 | 1.06 |
| Uterus | 0.18 | 1.05 | 0.95 | 0.11 |
| Total Body | 0.73 | 1.26 | 1.05 | 1.04 |
| **Average relative AD % difference*** | **-26%** | **34%** | **16%** | **6%** |
| ED | 0.59 | 1.23 | 0.84 | 0.54 |

*For each target organ, relative AD % difference for the gender average (GA) subject were calculated relatively to M1 according to the formula: ((AD(M#)-AD(M1)) /AD(M1))×100.

**Supplementary Table 4.** Extrapolated human absorbed dose (AD) and effective dose (ED) ratios for [^68^Ga]NODAGA-RGDyK.

| **^68^Ga-NODAGA-RGDyK** | **Human AD ratios**  **(GA subject)** | | | |
| --- | --- | --- | --- | --- |
| **Target Organ** | **M2/M1** | **M3/M1** | **M4/M1** | **M5/M1** |
| Adrenals | 0.62 | 2.00 | 1.51 | 0.79 |
| Brain | 1.11 | 3.64 | 4.38 | 2.23 |
| Breasts | 1.03 | 3.24 | 3.80 | 1.96 |
| Oesophagus | 0.91 | 2.69 | 2.91 | 1.51 |
| Eyes | 1.11 | 3.62 | 4.36 | 2.22 |
| Gallbladder Wall | 0.71 | 2.07 | 1.84 | 1.02 |
| Left colon | 0.32 | 2.43 | 0.95 | 0.36 |
| Small Intestine | 0.30 | 1.87 | 0.68 | 0.18 |
| Stomach Wall | 0.38 | 2.36 | 1.14 | 0.49 |
| Right colon | 0.32 | 2.41 | 0.94 | 0.37 |
| Rectum | 0.49 | 2.12 | 0.99 | 0.28 |
| Heart Wall | 0.85 | 2.47 | 2.38 | 1.04 |
| Kidneys | 0.33 | 2.16 | 0.72 | 0.35 |
| Liver | 0.57 | 1.14 | 0.67 | 0.56 |
| Lungs | 1.46 | 2.13 | 3.27 | 1.00 |
| Ovaries | 0.95 | 2.14 | 2.25 | 0.90 |
| Pancreas | 0.72 | 2.36 | 2.14 | 1.13 |
| Prostate | 0.98 | 1.84 | 1.95 | 0.67 |
| Salivary Glands | 1.11 | 3.60 | 4.34 | 2.21 |
| Red Marrow | 0.86 | 2.83 | 2.64 | 0.81 |
| Osteogenic Cells | 0.90 | 2.98 | 2.94 | 1.05 |
| Spleen | 0.59 | 1.37 | 0.84 | 0.26 |
| Testes | 1.06 | 2.70 | 3.15 | 1.43 |
| Thymus | 1.07 | 3.14 | 3.70 | 1.83 |
| Thyroid | 1.10 | 3.41 | 4.09 | 2.06 |
| Urinary Bladder Wall | 1.00 | 1.03 | 1.03 | 0.03 |
| Uterus | 0.95 | 1.69 | 1.71 | 0.53 |
| Total Body | 0.87 | 2.24 | 2.24 | 1.00 |
| **Average relative AD % difference*** | **-19%** | **142%** | **127%** | **1%** |
| ED | 0.80 | 1.58 | 1.26 | 0.32 |

*For each target organ, relative AD % difference for the gender average (GA) subject were calculated relatively to M1 according to the formula: ((AD(M#)-AD(M1)) /AD(M1))×100.

**Table 5**. Source organ AD extrapolations reassessed after the application of α_new_ .

|  | **AD ratios** | | | |
| --- | --- | --- | --- | --- |
| **Source Organ** | M3/H  (α=0.25) | M3/H  (α_new_=0.17) | M4/H  (α=0.25) | M4/H  (α_new_=0.17) |
| Left colon | 1.17 | 0.99 | 0.46 | 0.39 |
| Small Intestine | 1.04 | 0.93 | 0.38 | 0.33 |
| Stomach Wall | 1.32 | 1.13 | 0.64 | 0.54 |
| Right colon | 1.19 | 1.01 | 0.46 | 0.39 |
| Rectum | 1.21 | 1.05 | 0.56 | 0.5 |
| Heart Wall | 0.61 | 0.51 | 0.58 | 0.46 |
| Red Marrow | 1.12 | 0.94 | 1.05 | 0.88 |
| Spleen | 0.61 | 0.58 | 0.37 | 0.35 |
| Kidneys | 1.24 | 1.08 | 0.41 | 0.36 |
| Liver | 1.67 | 1.64 | 0.97 | 0.95 |
| Lungs | 0.59 | 0.51 | 0.91 | 0.79 |
| **Average of all source organs** | **1.07** | **0.94** | **0.62** | **0.54** |

The table compares [^68^Ga]NODAGA-RGDyK M3/H and M4/H obtained by applying α=0.25 and α_new_=0.17, respectively.
